# HC-Pro Disrupts miR319–TCP Regulatory Pathways to Induce Sterility in Transgenic Plants

**DOI:** 10.3390/ijms262110551

**Published:** 2025-10-30

**Authors:** Taicheng Jin, Weiyan Wang, Jiaxue Yu, Zhuyi Xiao, Yushuo Li, Xu Sun, Liping Yang

**Affiliations:** The School of Life Sciences, Jilin Normal University, Siping 136000, China; jintaicheng2535@163.com (T.J.); wwy98818@163.com (W.W.); 15330658182@163.com (J.Y.); 13086887230@163.com (Z.X.); m15568356195@163.com (Y.L.); sunxu1206@163.com (X.S.)

**Keywords:** HC-Pro, miR319, jasmonic acid pathway, TCPs, floral organ development

## Abstract

Helper component-proteinase (HC-Pro), encoded by *tobacco vein banding mosaic virus* (TVBMV), can cause various viral symptoms and even abortion. HC-Pro counteracts host-mediated inhibition by interfering with the accumulation of microRNAs (miRNAs) and small interfering RNAs (siRNAs). However, it is unclear whether the abortion phenotype of transgenic plants expressing HC-Pro is related to the abnormal expression of TEOSINTE BRANCHED 1/CYCLOIDEA/PROLIFERATING cell factors (TCPs), which are involved in regulating fertility. In this study, the molecular mechanisms through which HC-Pro causes various sterile phenotypes in plants were investigated. Reverse transcription–quantitative polymerase chain reaction (RT–qPCR) and Northern blotting revealed that in HC-Pro transgenic plants, the expression levels of *TCP4* and *TCP24* significantly increased. The increased expression of TCP4 further upregulated *LIPOXYGENASE2* (*LOX2*), a gene encoding a key enzyme in the synthesis of jasmonic acid (JA) precursors. Further studies confirmed that the aberrant expression of *TCP3*, *TCP4* and *TCP24* blocks the elongation of petals and anthers and that the aberrant expression of *TCP4* and *TCP24* blocks the release of pollen. This study demonstrated that HC-Pro affects the expression levels of the miR319-targeted genes *TCP2*, *TCP3*, *TCP4*, *TCP10* and *TCP24*, thereby affecting the normal development of floral organs and resulting in plant abortion. Both tobacco and *Arabidopsis thaliana* were used as model systems in this study on virus-mediated fertility, which provides important information for understanding how viral pathogenicity affects the regulation of fertility in crops.

## 1. Introduction

RNA silencing is a protective mechanism in plants that has developed over the course of evolution and plays important roles in maintaining genome stability and defending against invasion by exogenous viruses [1,2], and plant viruses encode silencing suppressors to counter this host defence mechanism [3]. Helper component-proteinase (HC-Pro), encoded by *Potato virus Y* (PVY), counteracts host inhibition by interacting with important functional proteins in host defence pathways [4,5]. Studies have shown that HC-Pro overexpression can cause severe viral symptoms in plants and potential developmental defects in floral organs, which can lead to plant abortion [6]. The most important plant immune hormones Salicylic acid (SA) plays an important in the process of pathogen defence. Our previous studies revealed that HC-Pro activates defence genes in the SA pathway by interfering in DNA methylation and induces the expression of *YUC* auxin synthesis genes, resulting in the increased local accumulation of auxin in plants and various abnormal growth and development phenotypes [7,8]. Further studies confirmed that Repressor of silencing 1 (ROS1)-mediated DNA demethylation plays important roles in regulating the expression of stress response genes and in response to biotic stresses [9]. The virulence protein P3 of *rice grassy stunt virus* (RGSV) activates somatic embryogenesis receptor kinase 4 (SERK4) by directly binding to its promoter and degrades RNA polymerase IV protein to suppress rice antiviral immunity [10]. Plants have evolved sophisticated mechanisms to balance growth and defence. The evolutionarily conserved polymerase-associated factor 1 complex (PAF1C) plays multiple roles in transcription, and regulates the growth–defence tradeoff by repressing defence genes expression in *Arabidopsis* [11].

The transcription factors TCP2, TCP3, TCP4, TCP10, and TCP24 are the main targets of miR319A and play important regulatory roles in plant leaf morphology, root growth, petal growth, plant fertility, and stress responses [12]. TCP4 regulates the jasmonic acid (JA) signalling pathway, thereby participating in the regulation of plant fertility [13,14]. Overexpression of the *TCP4* gene in plants can lead to severe floral organ defects and abortion [15]. The absence of key proteins in the JA biosynthetic or signal transduction pathways results in plant abortion [16]. TCP4 expression can increase the JA content. When the JA level is too high or too low, plants exhibit traits associated with abortion [17]. The cis-acting progenitor of LOX2, the gene encoding the key enzyme that synthesises the JA precursor, contains a binding site for TCP4, and their binding regulates plant fertility by affecting JA synthesis [12]. In *Arabidopsis thaliana*, ARF6 and ARF8 can activate the expression of *LOX2* and regulate inflorescence stem elongation, stamen filament elongation and pistil maturation [18]. TCP24 is a negative regulator of secondary cell wall thickening of the inner wall of the anther. Overexpression of the *TCP24* gene disrupts the thickening of the secondary cell wall of the inner wall of the anther, causing anther dehiscence and obstructing pollen release, eventually resulting in male sterility [19].

However, how HC-Pro affects sterility through TCP–miR319 regulation is not fully understood. We focused on the regulatory mechanism of gene expression that the viral suppressor HC-Pro resulted in plant abortion. This study revealed that HC-Pro affects the expression levels of the miR319-targeted genes *TCP2*, *TCP3*, *TCP4*, *TCP10* and *TCP24*, thereby affecting the normal development of floral organs. The floral organs of transgenic plants expressing HC-Pro were severely malformed or detached. In addition, the fertility-related genes *LOX2*, *ARF6*, and *ARF8* were significantly upregulated in transgenic plants, which was an important cause of abortion. Further studies confirmed that the *TCP2*, *TCP3*, *TCP4*, *TCP10*, and *TCP24* genes play important roles in regulating the elongation of petals and anthers and the release of pollen. The molecular mechanism underlying plant abortion caused by the viral suppressor HC-Pro was thus elucidated.

## 2. Results

### 2.1. Abnormal Development of Floral Organs in Transgenic Tobacco Plants Expressing HC-Pro

**Figure 1:**
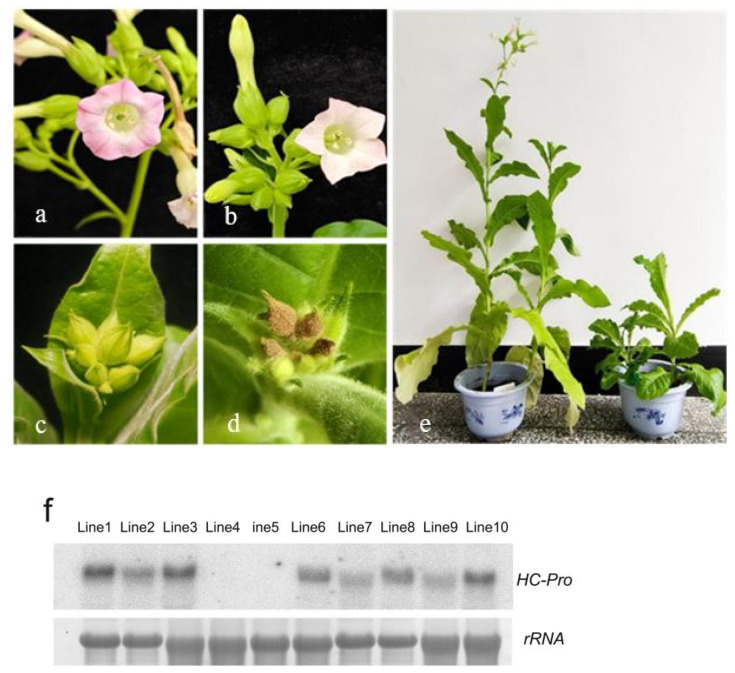
Sterility phenotype of floral organs and analysis of HC-Pro gene expression levels in HC-Pro transgenic lines. (**a**,**c**) Flower organs of control plants. (**b**) Flower organs of HC-Pro plants produced little pollen (**d**) Browning of floral organs of HC-Pro plants. (**e**) Left: control plants; right: stunted phenotype of the HC-Pro plants. (**f**) Northern blot analysis of the expression levels of the HC-Pro gene in the HC-Pro transgenic lines 1–10. Methylene blue-stained rRNA is shown as a loading control.

Transgenic tobacco plants were obtained via selection with kanamycin sulphate. The floral organs of tobacco plants transformed with the empty vector contained a large amount of pollen (Figure 1a). The flowers of the transgenic plants expressing HC-Pro produced little pollen (Figure 1b). Compared with those of the control plants (Figure 1c), the floral organs of the transgenic plants expressing HC-Pro were smaller, and their development was delayed. During development, many buds fell off, some buds turned brown (Figure 1d), and the flower buds closed without dehiscence, indicating complete abortion. Moreover, the transgenic plants expressing HC-Pro presented significant dwarf and late-flowering phenotypes (Figure 1e), their developmental cycle was significantly longer, and their flowering time was delayed by six months. In addition, compared with those of plants transformed with the empty vector, the leaf area of the transgenic plants expressing HC-Pro was reduced, the leaves were narrow and slender, and some leaves were severely curled and deformed.

The leaves of 10 transgenic tobacco plants with significant viral symptoms were selected for further analyses. Total RNA was extracted to determine gene expression levels. The transgenic tobacco plants were identified by Northern blotting. Compared with those in the other transgenic plants, the expression levels of the *HC-Pro* gene in Line 1, 3, 8 and 10 transgenic plants were greater (Figure 1f). The results revealed that the expression of *HC-Pro* results in severe developmental defects in floral organs and an abortion phenotype.

### 2.2. Abnormal Development of Floral Organs in Transgenic Tobacco Plants Expressing HC-Pro Is Associated with Abnormal Gene Expression

**Figure 2:**
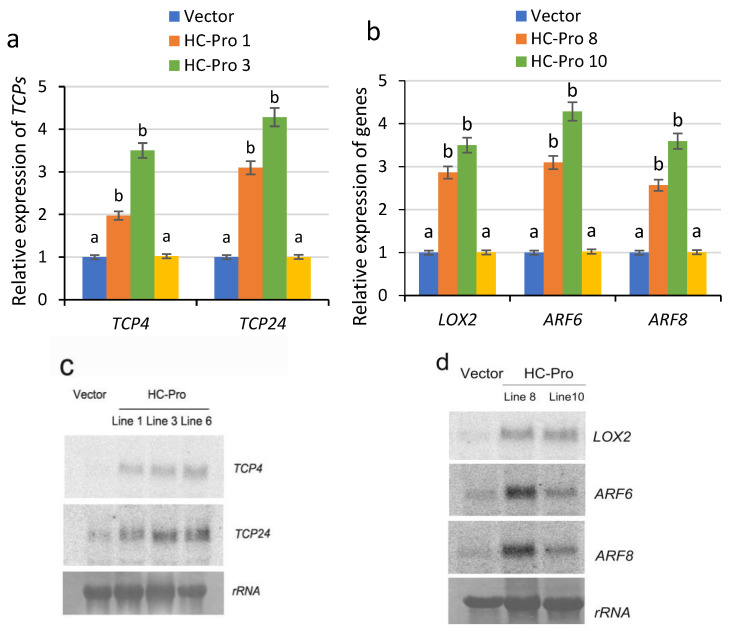
Analysis of the expression of genes related to fertility regulation in HC-Pro transgenic plants. (**a**,**c**) RT–qPCR and Northern blot analysis of the expression levels of *TCP4* and *TCP24* in HC-Pro transgenic plants, with expression in empty vector-transformed control plants used as controls. C2 represent the expression levels of of *TCP4* and *TCP24* in C2 transgenic plants, served as an additional negative control. (**b**,**d**) RT–qPCR and Northern blot analysis of the expression levels of *LOX2*, *ARF6* and *ARF8* in HC-Pro plants. C2 represent the expression levels of of *TCP4* and *TCP24* in C2 transgenic plants, served as an additional negative control. The RT–qPCR results were statistically analysed and plotted in Excel and GraphPad Prism 9.5 (two-way ANOVA, *p* < 0.05); the error bars represent the SEs from three biological replicates. Means identified by different letters are significantly different from each other. The error bars represent the SDs from three biological replicates.

To determine whether the abnormal phenotype of the floral organs of transgenic tobacco plants expressing HC-Pro is associated with the transcription factors TCP4 and TCP24, RT–qPCR and Northern blotting were used to assess the expression of related genes in the HC-Pro 1, HC-Pro 3 and HC-Pro 6 lines. The results revealed that the expression levels of *TCP4* and *TCP24* significantly increased in the transgenic tobacco plants expressing HC-Pro, with the gene expression levels in the empty vector-transformed plants used as controls (Figure 2a,c). Moreover, the increase in the expression levels of the *TCP4* and *TCP24* genes in HC-Pro transgenic Lines 1 and 3 was more significant, the floral organs presented severe browning, abscission, and abortion, and the HC-Pro transgenic plants presented a complete abortion phenotype. The differences in the expression levels of the HC-Pro gene in different transgenic plants resulted in different expression levels of the *TCP4* and *TCP24* genes, and these plants presented different abortion phenotypes.

To further explore the molecular mechanisms in transgenic tobacco plants expressing HC-Pro, the expression of the downstream genes *LOX2*, *ARF6*, and *ARF8*, which are regulated by TCP4, was analysed via RT–qPCR and Northern blotting. The results revealed that *LOX2*, *ARF6* and *ARF8* were significantly upregulated in the transgenic lines HC-Pro 8 and HC-Pro 10 (Figure 2b,d), confirming that the abnormal expression of these genes was the direct cause of various abortion phenotypes in transgenic plants.

### 2.3. Aberrant Phenotype and Identification of HC-Pro Transgenic A. thaliana

**Figure 3:**
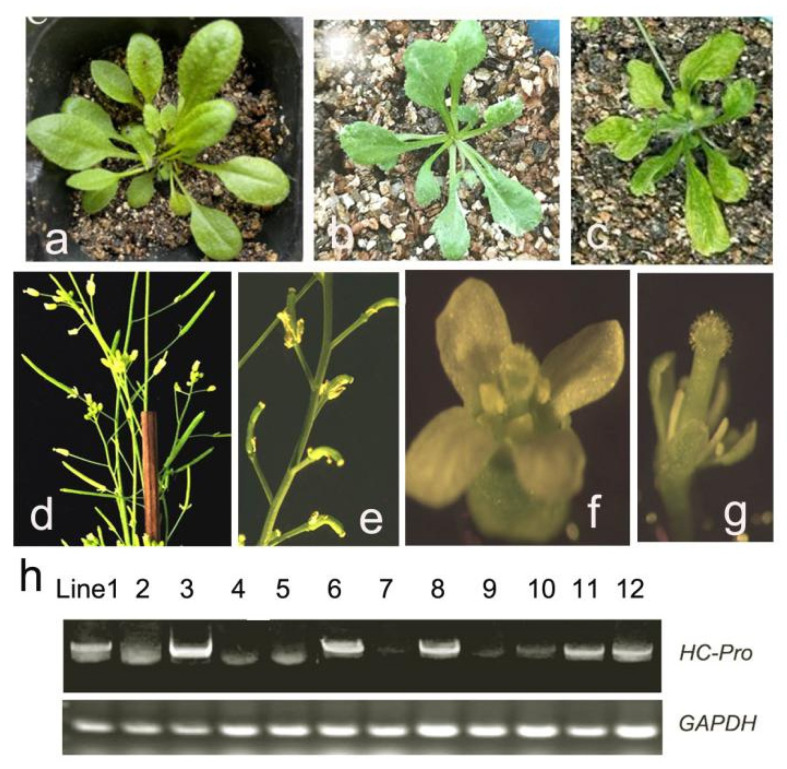
Observations of the phenotypes and identification of HC-Pro transgenic *A. thaliana*. (**a**) Control plants transformed with the empty vector. (**b**,**c**) Aberrant phenotype of the HC-Pro transgenic *A. thaliana*. (**d**) Fruit pods of plants transformed with the empty vector used as controls. (**e**) Shorter fruit pods in HC-Pro transgenic plants. (**f**) Floral organs of plants transformed with the empty vector used as controls. (**g**) Defects in floral organ development in the HC-Pro transgenic plants. (**h**) RT-sqPCR analysis of the expression levels of the HC-Pro gene in HC-Pro transgenic plants, with *GAPDH* used as a reference gene.

To elucidate the abortion mechanism in HC-Pro transgenic plants, transgenic *A. thaliana* plants were obtained via the floral dip method. The plants transformed with the empty vector served as controls (Figure 3a). The leaf area of transgenic *A. thaliana* expressing HC-Pro decreased, and the leaves were narrow and elongated (Figure 3b) and severely curled and deformed (Figure 3c). Compared with those of the control plants (Figure 3d), the fruit pods of the HC-Pro transgenic plants were shorter (Figure 3e). Transgenic *A. thaliana* plants expressing HC-Pro presented obvious abortion phenotype characteristics, including defects in floral organ development. Compared with the controls (Figure 3f), transgenic *A. thaliana* expressing HC-Pro presented smaller floral organs, very short anthers, and no pollen (Figure 3g).

Plants with these abnormal phenotypes were selected for analysis, and 12 transgenic plants expressing HC-Pro were identified. RT–qPCR revealed that the expression levels of the *HC-Pro* gene in the HC-Pro 2, HC-Pro 3, HC-Pro 6, and HC-Pro 8 transgenic plants were relatively high (Figure 3h). The higher the expression level of the exogenous gene HC-Pro was, the more obvious the abortion characteristics of the transgenic *A. thaliana* floral organ defects, with no production of fruit pods and complete abortion. When the expression level of the exogenous gene HC-Pro was low, the transgenic plants produced short and small pods, resulting in partial abortion.

### 2.4. Expression Analysis of Fertility-Related Genes in HC-Pro Transgenic A. thaliana

**Figure 4:**
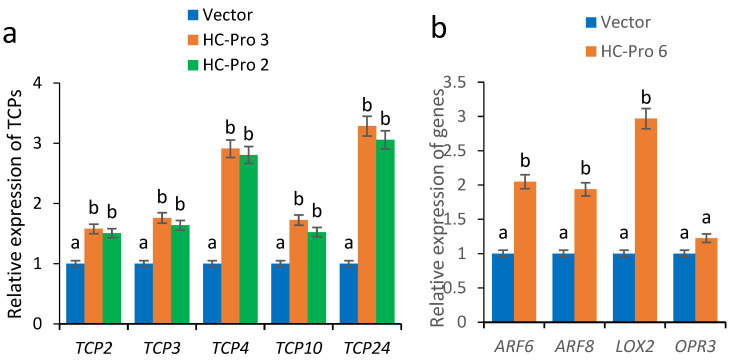
Expression analysis of fertility-related genes in HC-Pro transgenic *A. thaliana.* (**a**) RT–qPCR analysis of the expression levels of *TCP2*, *TCP3*, *TCP4*, *TCP10* and *TCP24* in the transgenic HC-Pro 2 and HC-Pro 3 lines. (**b**) RT–qPCR analysis of expression levels of *LOX2*, *ARF6*, *ARF8* and *OPR3* in the HC-Pro 6 transgenic line. Vector: expression levels of these genes in the plants transformed with the empty vector, used as controls. The RT–qPCR results were statistically analysed and plotted in Excel and GraphPad Prism 9.5 (two-way ANOVA, *p* < 0.05); the error bars represent the SEs from three biological replicates. Means identified by different letters are significantly different from each other. The error bars represent the SDs from three biological replicates.

In plants, the transcription factors TCP2, TCP3, TCP4, TCP10, and TCP24 are the main targets of miR319A and are involved in the regulation of plant leaf morphology and floral organ development [12,19,20]. To further study the correlation between the abortion phenotype of HC-Pro transgenic *A. thaliana* and the expression levels of the transcription factor TCPs, the expression levels of the TCPs were determined. Compared with those in *A. thaliana* transformed with an empty vector as controls, the expression levels of the *TCP2, TCP3, TCP4, TCP10* and *TCP24* genes significantly increased in the transgenic plants (Figure 4a). The abortion phenotype of the transgenic plants may be closely related to the abnormal expression of these genes. Moreover, the upregulation of *TCP4* and *TCP24* was most evident in the HC-Pro 3 transgenic line, which presented a complete abortion phenotype. Differences in the expression levels of HC-Pro caused differences in the expression levels of *TCP4* and *TCP24* in the various transgenic plants, and as a result, the abortion phenotypes of the transgenic plants significantly differed.

To further study the mechanisms of abortion in HC-Pro transgenic plants, the expression of the TCP4-regulated *LOX2* gene and other fertility-related genes was analysed. The results revealed that the expression levels of the *LOX2*, *ARF6*, and *ARF8* genes increased in the HC-Pro 6 transgenic plants. In particular, *LOX2* was significantly upregulated, whereas the expression level of the OPR3 gene did not change significantly (Figure 4b). We speculated that the upregulation of TCP4 further promotes *LOX2* expression and that the increased expression of these fertility-related genes leads to multiple abortion phenotypes in plants.

### 2.5. Recovery of Fertile Phenotypes in Plants Harbouring the miR319abc Deletion Mutant

**Figure 5:**
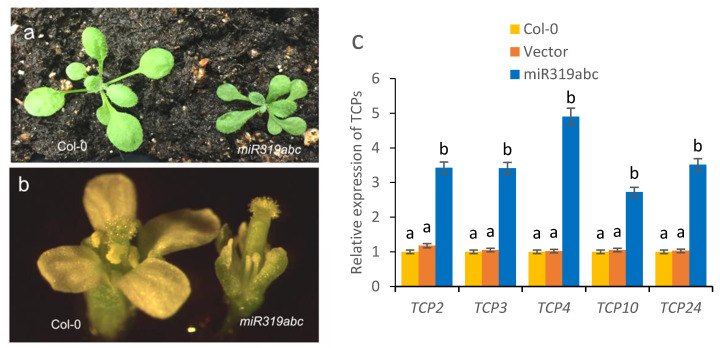
Phenotype of the miR319abc triple mutant and *TCP* expression analysis. (**a**) Seedling phenotype of the triple mutants of miR319abc compared with wild-type Col-0 and vector control. (**b**) Flower phenotype of the miR319 triple mutants compared with the floral organs of the Col-0 control. (**c**) The expression levels of *TCP2*, *TCP3*, *TCP4*, *TCP10* and *TCP24* in the miR319abc triple-mutant plants are relative to their expression in Col-0 and vector control plants. The RT–qPCR results were statistically analysed and plotted in Excel and GraphPad Prism 9.5 (two-way ANOVA, *p* < 0.05); the error bars represent the SEs from three biological replicates. Means identified by different letters are significantly different from each other. The error bars represent the SDs from three biological replicates.

miR319 genes, including miR319A, miR319B, and miR319C, are functionally redundant, with miR319A as the main gene, and play important roles in the regulation of plant growth and development [12]. CRISPR/Cas9 gene editing technology was used to obtain the mutant miR319a, miR319b and miR319c, and plant hybridization was used to obtain the miR319abc triple mutant, and the phenotype of these plants was very similar to that of HC-Pro transgenic *A. thaliana*, including smaller plants, narrow and elongated leaves (Figure 5a), severe floral organ defects, short anthers, and no pollen (Figure 5b), thus exhibiting a complete abortion phenotype. However, single mutation of miR319a yielded plants that produced a small amount of pollen, resulting in a partial abortion phenotype. Since *TCP2*, *TCP3*, *TCP4*, *TCP10*, and *TCP24* are the target genes of miR319A/JAW, the expression levels of these genes in the miR319abc triple-mutant plants were analysed, and the results revealed that the expression levels of *TCP2*, *TCP3*, *TCP4*, *TCP10*, and *TCP24* significantly increased (Figure 5c). The results also demonstrated that HC-Pro affected the expression levels of the miR319-targeted TCP transcription factors (TCP2, TCP3, TCP4, TCP10 and TCP24), thereby affecting the normal development of floral organs and resulting in plant abortion.

To further study the correlation between these abortion phenotypes and the expression levels of *TCP2*, *TCP3*, *TCP4*, *TCP10*, and *TCP24*, the *TCP2*, *TCP3*, *TCP4*, *TCP10*, and *TCP24* genes, respectively, were knocked out in miR319abc triple-mutant plants. The important role of these genes in the regulation of plant fertility was analysed. The petals of the *TCP3-*, *TCP4-* and *TCP24*-knockout plants were clearly longer, and the floral organ defect phenotype was significantly restored, pollen was produced, and fertility was partially restored in the *tcp4tcp24* double-mutant plants. The *tcp3tcp4tcp24* triple-mutant plants exhibited a completely restored floral organ defect phenotype, and their fertility was also completely restored (pollens and seeds were qualitatively observed). These results show that the *TCP2*, *TCP3*, *TCP4*, *TCP10* and *TCP24* genes play redundant functional roles in the regulation of floral organ development.

## 3. Discussion

### 3.1. Abortion in HC-Pro Transgenic Plants Is Associated with Abnormal Expression of Fertility-Related Genes

Specific gene silencing is a regulatory mechanism that is mediated by small RNAs, among which siRNAs and miRNAs play important roles in these regulatory processes [21]. The gene silencing suppressors encoded by viruses counteract host inhibition by interacting with the accumulated siRNAs [4,5]. Our previous study revealed that the viral suppressor HC-Pro from *tobacco vein banding mosaic virus* (TVBMV) interferes with the accumulation of siRNAs and affects the expression of plant SA pathway defence genes [8]. The gene silencing suppressor P1/HC-Pro encoded by *turnip mosaic virus* (TuMV) interferes with miR171-mediated nucleolytic activity, thereby inhibiting the miR171s-targeted cleavage of Scarecrow-like transcription factors and enabling systemic plant viral infection [6]. In this study, we elucidated the molecular mechanisms by which HC-Pro causes various sterile phenotypes in plants and demonstrated that the increased expression of the fertility-related genes *LOX2*, *ARF6* and *ARF8* leads to multiple abortion phenotypes in HC-Pro transgenic plants.

The overexpression of the *TCP4* gene in *A. thaliana* significantly reduces fertility, and most floral organs fell off [22]. In the JA pathway, the downstream gene of TCP4 is *LOX2*, which encodes a chloroplast enzyme and promotes JA synthesis in sepals and stamens [23]. In addition, excessive activation of *TCP4* can lead to reduced cell proliferation, which in turn leads to smaller leaves [24]. TCP24 is an important regulatory factor of secondary wall thickening in *A. thaliana*. The higher the expression level of TCP24 is, the greater the degree of male sterility [25]. In this study, the expression levels of *TCP4* and *TCP24* significantly increased in transgenic *A. thaliana* plants expressing HC-Pro (Figure 4a), which produced short fruit pins (Figure 3) and short anthers. We speculated that the increased expression of *TCP4* further affected the JA content, resulting in defects in floral organ development. The anthers did not produce pollen, indicating obvious male abortion, which was speculated to be related to the increased expression of *TCP24*.

### 3.2. The Viral Suppressor HC-Pro Interferes with miR319–TCP Regulatory Pathways

MiR319A/JAW negatively regulates the transcription of TCP transcription factors, particularly TCP4. This disrupts floral organ development, ultimately impairing plant fertility [14]. This study confirmed that the expression levels of *TCP4* and *TCP24* significantly increased in transgenic *A. thaliana* expressing HC-Pro; moreover, the expression levels of *TCP2*, *TCP3* and *TCP10* also significantly increased (Figure 4a). We speculated that HC-Pro repressed the accumulation of miRNAs from the miR319 gene, thereby increasing the expression levels of *TCP2*, *TCP3*, *TCP4*, *TCP10*, and *TCP24*. Further studies confirmed that the *miR319abc* triple-mutant plants present severe defects in floral organs, have short anthers (Figure 5b), cannot produce pollen, and exhibit an obvious abortion phenotype, which is closely related to the increased expression levels of *TCPs* in these plants (Figure 5c). *TCP2*, *TCP3*, *TCP4*, *TCP10*, and *TCP24* were knocked out in *miR319abc* triple-mutant plants, confirming the important role of these genes in regulating the normal development of floral organs. The simultaneous knockout of the *TCP4* and *TCP24* genes significantly restored the floral organ defect phenotype and partially restored fertility. Simultaneous knockout of the *TCP3*, *TCP4*, and *TCP24* genes completely restored the floral organ defect phenotype, and fertility was completely restored. These results indicate that these genes play redundant functional roles in the regulation of floral organ development. In addition, we revealed that the *TCP4* and *TCP24* genes play important roles in the regulation of anther elongation and pollen production and release.

Specific TCP family members serve as key factors that regulate crop fertility, influence ornamental value, and affect crop yield by influencing the morphology and structure of floral organs [26]. Moreover, TCP transcription factors exert their effects by mediating complex regulatory networks. In addition to their ability to control growth and development, they participate in the regulation of hormone responses and stress responses [27]. Our findings have a potential broader implications for the manipulation of TCP activity. These findings open new avenues for research into the involvement of *TCP* in the regulation of crop fertility and disease resistance or in the breeding of stress tolerance.

## 4. Materials and Methods

### 4.1. Agrobacterium-Mediated Leaf Disc Transformation of Tobacco (Nicotiana tabacum L.)

Sterile seedlings were selected, and the leaves were cut and inoculated in premedium for 2 days. *Agrobacterium* strain EHA105-pBI121-HC-Pro was activated, cultured, and harvested. MMA was added, and the *OD*_600_ of the resuspended culture was adjusted to 0.3–0.5. The leaves were placed in the bacterial mixture for 15 min and then transferred to coculture medium and cultured in the dark for 2 days. Next, the leaves were transferred to callus induction medium and cultured for 30 days, the calli were induced to grow clustered buds, and the leaves were subsequently transferred to selection medium. The seedlings in the selected medium were transferred to rooting medium to complete rooting culture. HC-Pro transgenic tobacco plants have two sterility phenotypes, partial sterility (ability to harvest a small number of seeds) and complete sterility (the plant does not produce seeds).

### 4.2. Northern Blotting Identification of HC-Pro Transgenic Plants

For high-molecular-weight RNA gel analysis, 10 mg of total RNA from HC-Pro transgenic tobacco plants was extracted via the TRIzol method, separated on 1% agarose–formaldehyde gels, transferred to Hybond-Nþ membranes, and hybridised with DIG-labelled probes following the DIG system’s user guide (Roche, Shanghai, China). The biological experiments were independently repeated three times. For details, please refer to our previous study [8].

### 4.3. RT–qPCR Analysis of the Expression of Relevant Genes in HC-Pro Transgenic Plants

Total RNA from transgenic tobacco plants expressing HC-Pro was extracted and subjected to reverse transcription using a kit to prepare cDNA. All cDNA samples were prepared in separate reaction systems. Real-time PCR was performed for each sample for the *LOX2*, *ARF6*, and *ARF8* target genes and the internal reference, and three wells were used for each sample. The biological experiments were independently repeated three times. One-way ANOVA followed by Tukey’s multiple comparison test was used for statistical analysis according to our previous method [28].

### 4.4. Obtaining Mutants via CRISPR/Cas9 Genome Editing

The triple-mutant miR319abc and the knockout vectors for *TCP2*, *TCP3*, *TCP4*, *TCP10*, and *TCP24* were kindly donated by Professor Yunde Zhao, Nanjing University. Triple-mutant miR319abc was used as the plant material, and the TCP2, TCP3, TCP4, TCP10, and TCP24 genes were knocked out via CRISPR/Cas9 genome editing. The *tcp2, tcp3*, *tcp4*, *tcp10,* and *tcp24* mutants were screened via PCR, and the double mutants *tcp2 tcp3*, *tcp3 tcp24*, and *tcp4 tcp24* and the triple mutant *tcp3 tcp4 tcp24* were obtained by crossbreeding.

## 5. Conclusions

**Figure 6:**
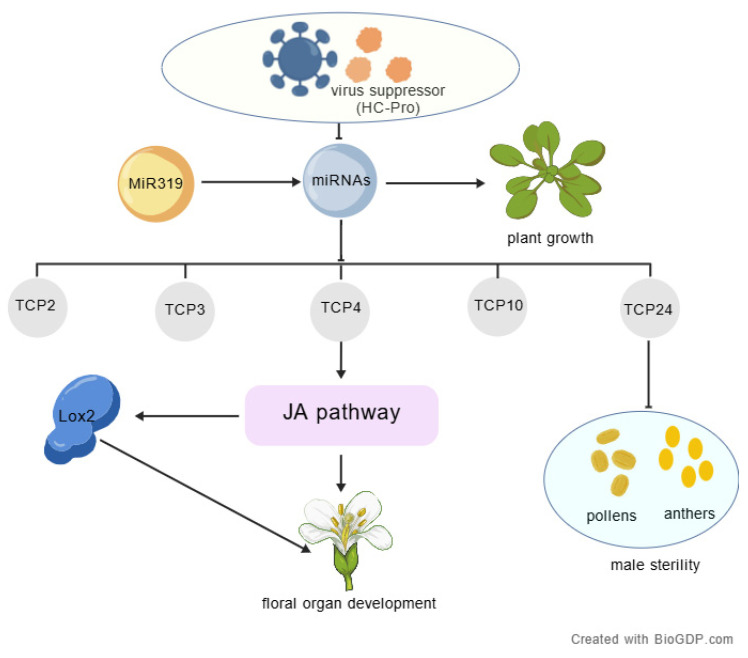
HC-Pro disrupts miR319–TCP regulatory pathways to induce sterility in plants.

In this study, *Agrobacterium*-mediated genetic transformation was used to obtain transgenic tobacco and transgenic *A. thaliana* plants expressing HC-Pro. The results revealed that the miR319-targeted genes *TCP2*, *TCP3*, *TCP4*, *TCP10* and *TCP24* were significantly upregulated in the transgenic plants expressing HC-Pro. In addition, the expression levels of the fertility-related genes *LOX2*, *ARF6*, and *ARF8* significantly increased, resulting in the abortion of the transgenic plants. Further studies confirmed that the *TCP4* and *TCP24* genes play important roles in regulating the elongation of petals and anthers and the release of pollen. This study revealed that HC-Pro likely disrupts miR319–TCP regulatory pathways by interfering with miR319 accumulation, thereby affecting normal floral organ development and contributing to sterility phenotypes (Figure 6).

## Data Availability

The original contributions presented in this study are included in the article. Further inquiries can be directed to the corresponding author.
